# Effects of lupeol and flutamide on experimentally-induced polycystic ovary syndrome in mice

**DOI:** 10.22038/IJBMS.2024.77602.16783

**Published:** 2024

**Authors:** Ali Rezaei-Golmisheh, Rajabali Sadrkhanlou, Abbas Ahmadi, Hassan Malekinejad

**Affiliations:** 1 Department of Basic Sciences, School of Veterinary Medicine, Ardakan University, Ardakan, Iran; 2 Biology and Animal Reproduction Science Research Institute, Ardakan University, Ardakan, Iran; 3 Department of Comparative Histology and Embryology, Faculty of Veterinary Medicine, Urmia University, Urmia, Iran; 4 Department of Anatomy, Faculty of Veterinary Medicine, Urmia University, Urmia, Iran; 5 Department of Pharmacology and Toxicology, School of Pharmacy, Urmia University of Medical Sciences, Urmia, Iran

**Keywords:** Anti-androgen, Atretic follicles, Fertility rate, Fibrosis, In vitro fertilization

## Abstract

**Objective(s):**

Polycystic ovary syndrome (PCOS) is one of the main causes of infertility in women. This study was conducted to uncover the effects of lupeol as an anti-androgenic triterpene on experimentally-induced PCOS in mice.

**Materials and Methods:**

Eighty immature female mice were divided into 4 groups: Control (C), PCOS (P), Lupeol (L), and Flutamide (F). PCOS was induced in test groups by injection of Dehydroepiandrosterone (60 mg/kg/day, IP) for twenty days. Following the PCOS induction, the two groups of L and F were treated with lupeol (40 mg/kg/day) and/or flutamide (10 mg/kg/day) respectively and the two groups of C and P received sesame oil (0.1 ml/mouse/day) for 15 days. After the treatment period, ten animals in each group were selected for collecting blood and ovary samples.* In vitro * fertilization assessment was carried out on 10 remaining mice in each group. The hormonal assays and oxidative stress biomarker determination were performed on serum and tissue samples. Moreover, histopathological analyses were conducted on the ovaries.

**Results:**

PCOS-elevated concentration of LH and Testosterone was significantly (*P*<0.05) lowered in lupeol and flutamide-received animals. Lupeol and flutamide both reduced PCOS-induced fibrosis and the number of atretic follicles. Both compounds declined the PCOS-increased lipid peroxidation and protein oxidation in serum and the ovaries. Lupeol increased the PCOS-reduced fertility rate and decreased the number of arrested embryos by 12%.

**Conclusion:**

These findings indicate that lupeol could be a novel compound in the treatment of PCOS as it reduced PCOS-induced structural and also functional disorders.

## Introduction

Polycystic ovary syndrome (PCOS) is the most prevalent endocrine disorder of women of reproductive age which affects 6–15% of women in different communities (1, 2). It is considered the major cause of infertility because of the induction of anovulation and hyperandrogenism in young women (3). Although the initiator factor for PCOS has not been definitely identified, theca-cell hyperplasia has been suggested as one of the underlying causes and the cause of hyperandrogenism and polycystic ovaries (4). Recent findings suggest that androgens can inhibit the expression of progesterone receptors and impede its negative feedback by binding to the androgen receptors in the hypothalamus (5, 6) thereby increasing the GnRH pulsatile secretion and subsequently LH secretion from the pituitary (7). These changes in the secretion of gonadotropins can impair follicular development and ovulation. Arrested follicles with cystic structures result in hyperandrogenemia and loss of fertility in PCOS patients (4).

Relationship between theca cell hyperplasia and insulin resistance, hyperinsulinemia, and increased insulin-like growth factors has been previously reported (8-10). Metformin as an insulin-sensitizing drug has beneficial effects in reducing hyperinsulinemia, improving lipid profile, and increasing ovulation in patients with PCOS, but it is less effective in improving clinical hyperandrogenism and hirsutism (11).

Regarding the crucial role of hyperandrogenism in the induction of PCOS, different medicines with anti-androgenic effects such as Spironolactone (SPL), Cyproterone acetate (CPA), Flutamide, and/or Finasteride have been used in therapeutic protocols. Flutamide is a synthetic non-steroidal inhibitor of androgen receptors (12) that decreases androgen levels in the bloodstream by reducing the synthesis of androgens and increasing their metabolism. Flutamide treatment reduces hirsutism (13, 14) and decreases plasma levels of LH, proportional level of LH/FSH, androgens, triglycerides, and LDL levels in young girls and women with hyperinsulinemic hyperandrogenemia or PCOS (15-17), but its effects in restoring ovulatory cycles and menstruation in anovulatory PCOS patients is a matter of debate (16, 17). Flutamide treatment has side effects that mostly include: digestive system problems, diarrhea, and liver toxicity (18, 19), however, because of its non-steroidal structure, it has lower hormonal side effects than CPA, and unlike SPL does not affect kidney function; however, it should be given with a lower dose and more carefully in long term because of rare complications of mild hepatotoxicity (14, 20).

There is a growing tendency to use natural triterpenoids as phytosterols, due to the wide range of their biological activities (21). Triterpenoids are a large group of natural compounds, which stabilize the phospholipid bilayers of cell membranes (22). In human diets, triterpenoids are mainly derived from cereals, vegetables, fruits, and plant-derived oils (23). Lupeol is a triterpenoid that has recently gotten the attention of researchers and medical professionals (24). Lupeol is found in cabbage, pepper, olive, mango, red grapes, ginseng, and some other vegetables, fruits, or medicinal plants (25). A wide range of pharmacological effects of lupeol has been shown in *in vitro* and *in vivo* models including beneficial effects on inflammation, cancer, arthritis, diabetes, heart disease, metabolic disorders including non-alcoholic fatty liver disease, polycystic ovarian syndrome-related inflammation, and renal and hepatic toxicity (25-33). Due to the structural similarity of lupeol to androgens, the effects of lupeol on AR signaling have been studied and anti-androgen receptor activity of lupeol has been previously highlighted. A competitive antagonism has been documented as the mechanism of action of lupeol (34, 35).

The approved anti-androgenic effects of lupeol on the one hand and the crucial role of androgens in the pathophysiology of PCOS on the other hand, directed us to investigate the effects of lupeol compared to flutamide on the dehydroepiandrosterone-induced PCOS in mice. The potential effects of lupeol on PCOS-induced alterations in the structure and function of ovaries along with its effect on the rate of *in vitro* fertilization were investigated. 

## Materials and Methods

***Chemicals***

Dehydroepiandrosterone (D4000) and Lupeol (L5632) were procured from Sigma, USA. Flutamide (Flutan) was purchased from Medochemie, Cyprus. A common type of edible sesame oil was obtained from a local grocery. PMSG and hCG hormones were purchased from Folligon, the Netherlands. The hematoxylin and eosin (H&E) and AZAN staining kits were supplied by Asia Pajohesh, Iran. Drugs were dissolved in sesame oil on a daily basis and used freshly.

***Animal grouping and treatments***

Eighty immature 20-day-old weaned female NMRI mice weighing approximately 14–17 g were obtained from the Laboratory Animal Center of the Faculty of Veterinary Medicine, Urmia University, Iran. Mice were housed in standard polycarbonate cages in a room with controlled temperature at 23–24 °C and continuous air renovation on a 10:14 hr light-dark cycle and access to regular chow diet and fresh tap water* ad libitum*.

Animals were randomly divided into 4 groups of twenty mice in each group. The groups were nominated as Control (C), PCOS (P), Lupeol (L), and Flutamide (F) and treatment of animals started at the age of 22 days after 2 2-day adaptation periods to the environmental conditions. For induction of PCOS, test groups including the P, L, and F received DHEA (60 mg/kg/day, IP), and the control group received 0.1 ml/mouse/day sesame oil (SO) for twenty consecutive days (36). Afterward, the two groups of L and F were treated with lupeol (40 mg/kg/day) (35) and/or Flutamide (10 mg/kg/day) respectively, and the two groups of C and P received SO (0.1 ml/mouse/day) for 15 consecutive days. All medications were administered intraperitoneally and sesame oil was used as drug solvent and vehicle. Finally, the estrus cycle was determined by preparing vaginal smears and cytological staining of smears using crystal violet. Ten animals in each group in the metestrus/diestrus phase were selected for collecting blood and ovary tissue samples. To evaluate the fertility potential of the animals, an *in vitro* fertilization experiment was carried out on 10 remaining mice in each group.

The Animal Care and Use Committee at Urmia University approved the experimental procedures on animals (IR-UU-AEC/1395/89/2d).

***Sampling***

After induction of deep general anesthesia by IP injection of 0.1 ml/20 g B.W. of a Ketamine and Xylazine cocktail (17.5 mg/ml Ketamine/2.5 mg/ml Xylazine), blood samples were collected via cardiac puncture and were centrifuged at 1500 ×g for 10 min to separate blood serum. Collected serum from each animal was aliquoted into 100 μl volumes and stored at -20 °C until further analyses were performed. The ovaries were dissected out and cleaned with chilled saline normal solution. Thereafter, for all animals, the right ovary was snap frozen in liquid nitrogen and stored at -80 °C and the left ovary was fixed in 10% formalin solution and stored at room temperature until being processed for histology analyses.

***Sex hormone levels in serum***

The serum level of LH, FSH, Estradiol (E2), and Testosterone (T) hormones were determined in serum samples using commercially available ELISA kits (Monobind Inc, Lake Forest, CA, USA) according to the provided instructions by the manufacturers.

***Histological analyses of ovaries***

Formalin-fixed ovarian tissues were dehydrated by passing a standard graded series of ethanol and cleared with xylene, embedded in paraffin, and finally serially sectioned into 5 µm sections. To stain the prepared sections, H&E and/or Azocarmine-Aniline blue (AZAN) methods were performed. Histomorphometric study of the ovaries was performed with an optical microscope (Olympus Bx50) to determine the growth and atresia of ovarian follicles and counting follicles in different developmental stages in H & E stained ovarian serial sections (37, 38).

The qualitative study of collagen changes and tissue fibrosis in the ovaries (39) was performed on AZAN-stained sections (40). Hence, the blue areas were considered as collagen fiber sedimentation and quantitative measurements of collagen sedimentation and fibrosis were performed by image analysis using Matlab software (V. 8.1.0) in 40 microscopic images from each group. The average ratio of blue pixels to the entire image pixels was determined as the mean percentage of tissue collagen (41).

***Oxidative stress assessment***

*Total anti-oxidant capacity (TAC) of ovarian tissue and serum*

The total anti-oxidant capacity of ovarian tissue and serum samples was measured using the FRAP method. In this method, in acidic pH which has been created by acetate buffer, the blue color produced by the reduction of Fe^3+^ ions of the Fe (III)-TPTZ complex and converting them into ferrous (Fe^2+^) ions is measured at 593 nm by a spectrophotometer (42).

***Malondialdehyde (MDA) levels in ovarian tissue and serum***

To determine the rate of lipid peroxidation, the concentration of malondialdehyde in ovarian tissue and serum samples was measured by thiobarbituric acid (TBA) reaction. Each single ovarian specimen was separately homogenized in a cold potassium chloride solution (150 mM), and the resulting mixture was centrifuged at 3000 rpm for 10 min. For each ovary specimen, 500 μl of supernatant and for serum samples, 200 μl of serum were mixed with 3 ml phosphoric acid (1% v/v), vortexed, and then 1 ml of TBA (6/7 g/l) was added. The samples were placed at 100 °C for 45 min and then cooled in ice. Finally, after adding 3 ml of n-butanol the samples were centrifuged again at 3000 rpm for 10 min. The absorbance of supernatant was measured at 532 nm by a spectrophotometer. The standard calibration curve of malondialdehyde was used for the calculation of the produced MDA level and expressed as nanomoles of malondialdehyde per mg of protein (43). The protein content of the samples was also measured according to the Lowry method (44).

***Protein carbonylation (CO) in ovarian tissue and serum***

The level of protein oxidation in ovarian tissue as well as serum samples was determined by measuring the amount of carbonyl content. The reaction of 2, 4-dinitrophenylhydrazine (DNPH), and carbonyl groups of proteins was measured based on a previously published method (45). Briefly, each ovary specimen was first homogenized in a cold phosphate buffer (50 mM, pH 7.6, containing EDTA 1 mM), and the resulting mixture was centrifuged at 10,000 × g and 4 °C for 10 min. For each ovary specimen, 200 μl of supernatant, and for serum samples, 200 μl of serum were used; one sample as test (T) and one sample as control (C) were prepared. Thereafter 800 μl of DNPH and hydrochloric acid solution (2 M) were added to the test and control samples, respectively. Samples were kept in a dark place at room temperature for one hour and vortexed every 15 min. Then, 0.5 ml of trichloroacetic acid (30%) was added to each sample and vortexed for 30 sec. All samples were centrifuged for 10 min at 10,000 ×g for 3 min, the supernatant was discarded and residual sediment was suspended in 1 ml ethanol/ethyl acetate solution (1:1) for 15 min. After centrifugation at 10,000 ×g for 3 min and dispersing the supernatant, the above step was repeated. After the last washing, the precipitate was dissolved in 0.6 ml of guanidine hydrochloride solution (6 M) at 37 °C for 15 min and then the samples were centrifuged for 10 min at 10,000 ×g for isolating and depositing any residues. For each sample, the optical density (OD) of the control and test was measured against a solution of guanidine hydrochloride (6 M) at a wavelength of 370 nm. The carbonyl content was determined as follows: 

Carbonyl (nmol/ml) = [(CA)/ (0.011 mM^-1^)] (600 µl/200 µl)

Where CA is the corrected absorbance and computed as the average OD for each control sample was subtracted from the average OD of the test sample at 370 nm. The extinction coefficient for DNPH at 370 nm is 22,000 M^-1^cm^-1^.

***In vitro fertilization (IVF)***

Ten remaining animals in each group were subjected to hormonal treatment according to a well-established super-ovulation hormonal regimen (45, 46). At the final hr of the 35^th^ day of the treatment period, animals received 10 IU PMSG (Folligon, The Netherlands) per capita, and 46–48 hr later, were injected with 10 IU of hCG (Folligon, The Netherlands) for induction of ovulation.

The next morning (10–12 hr after injection of hCG), one healthy young male mouse for three female mice were selected and after euthanizing by cervical dislocation, the abdominal region was completely sterilized and both epididymides were pulled out through a small cut on the scrotum. Epididymis was placed in a petri dish containing 1 ml HTF medium (CO_2_ pre-incubated and containing 4 mg/ml BSA) and a few cuts were made by surgical blade to let the sperms swim out. The sperms obtained their capacitation after about one hour of incubation.

To obtain oocytes, female animals were euthanized, their abdominal region sterilized, and the oviducts were isolated through an incision on the abdomen and placed in a petri dish containing pre-incubated (37 °C) and pH-equilibrated HTF medium. Using fine point gauge 30 needles and a dissecting technique, the oocytes were pulled out of the ampulla. Using a mouth pipette, the oocytes were washed in a series of medium droplets underneath the mineral oil and finally transferred to the droplets of the fertilization medium. Thereafter capacitated sperms (one million/ml in fertilization medium) were introduced to the oocytes and incubated until completion of fertilization. After about 12 hr, pair pronuclei were seen and then fertilized zygotes were picked up and washed a few times and cultured for 120 hr in 100 μl droplets under mineral oil (47). To evaluate embryonic development, the number of two-cell embryos after 24 hr, the number of blastocysts, and the arrested embryos in each group after 120 hr of incubation were recorded. The type of embryos that failed to develop was determined based on the following criteria:

Type I: Embryos with lysis, fragmentation, and complete necrosis;

Type II: Embryos with lysis and fragmentation in a number of blastomeres 

Type III: Embryos with a small number of lysed, fragmented blastomeres, and cytoplasmic vesicles.

***Statistical analysis***

Data were analyzed using SPSS software v.20 and the results were expressed as mean ± SD. In order to compare the results, one-way ANOVA and Tukey’s *post hoc* analysis were performed. The results of IVF tests were analyzed using Minitab software v.16 and 2 proportion statistical tests. The *P*-value (*P*<0.05) was considered to be statistically significant.

## Results

***The stage of estrous cycle was determined by vaginal smear***

**Figure 1:**
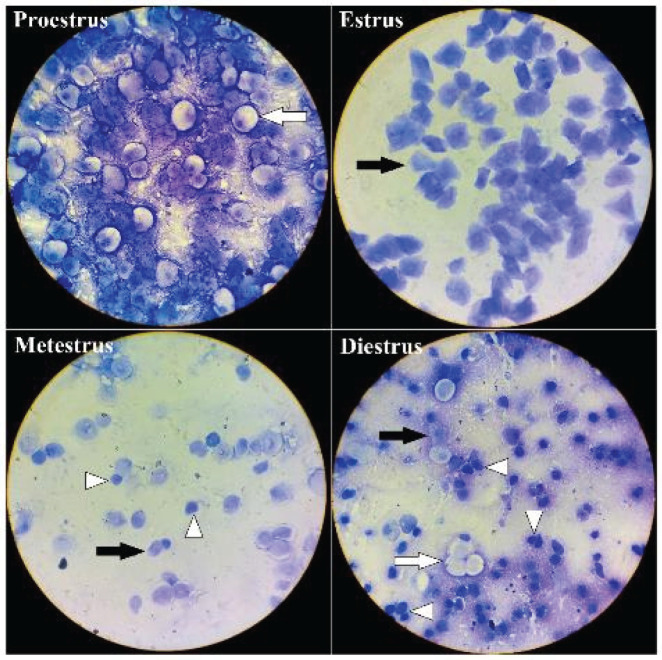
Vaginal smear of mice showing different cell types in four stages of estrus cycle

To identify the stage of the estrous cycle of animals, the relative ratio of cell types in smears was used. Three primary cell types that were detected in vaginal smear samples are nucleated epithelial cells (lightly stained cytoplasm with an oval nucleus), cornified squamous epithelial cells (anucleated uniformly stained polygonal cells), and polymorphonuclear leukocytes (irregularly shaped, small-sized cells with darkly stained polymorphic nuclei) (Figure 1). During the proestrus stage, cells were almost exclusively nucleated epithelial cells, and in the estrus stage, cells were predominantly squamous cornified epithelial cells, forming densely packed clusters. During metestrus, squamous cornified epithelial cells were still observed but small and darker leukocytes were also present, and in the diestrus stage there were a few if any squamous cornified epithelial cells remaining, leukocytes were present in large numbers, and nucleated epithelial cells were found as single cells or in clusters.

***Lupeol declined the PCOS-elevated level of LH and testosterone in serum***

**Figure 2:**
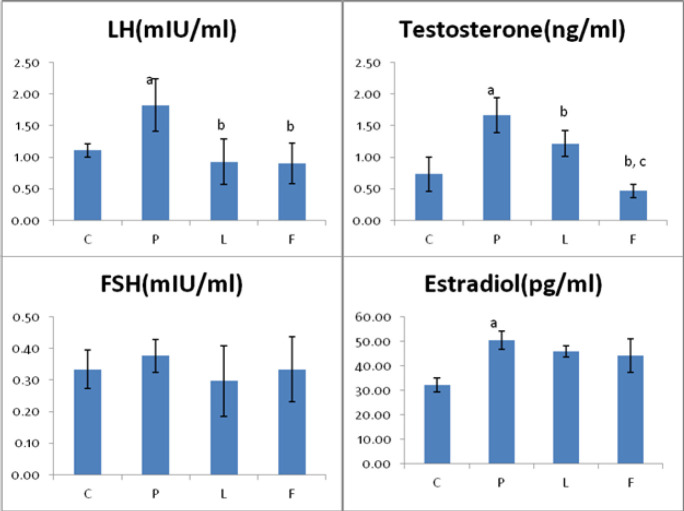
Concentrations of sex hormones LH, FSH, Estradiol (E2) and Testosterone (T) in serum of Control (C), PCOS (P), Lupole (L) and Flutamide (F) received mice

The concentrations ​​of LH, FSH, Estradiol (E2), and Testosterone (T) in the blood serum of animals are shown in Figure 2. The results are indicating a significant (*P*<0.05) increase in the concentration of LH, estradiol, and testosterone in group P compared to group C. Although the FSH level showed a slight increase, it was not significant when compared to the control animals. Lupeol and flutamide both were able to reduce the PCOS-increased level of LH, testosterone (significantly), FSH, and estradiol (non-significantly). We failed to show any significant difference between groups L and F, except their effect on testosterone levels, in which flutamide showed a dominant and significantly (*P*<0.05) more testosterone reduction than lupeol. 

***Lupeol reduced the PCOS-induced number of atretic follicles***

**Figure 3:**
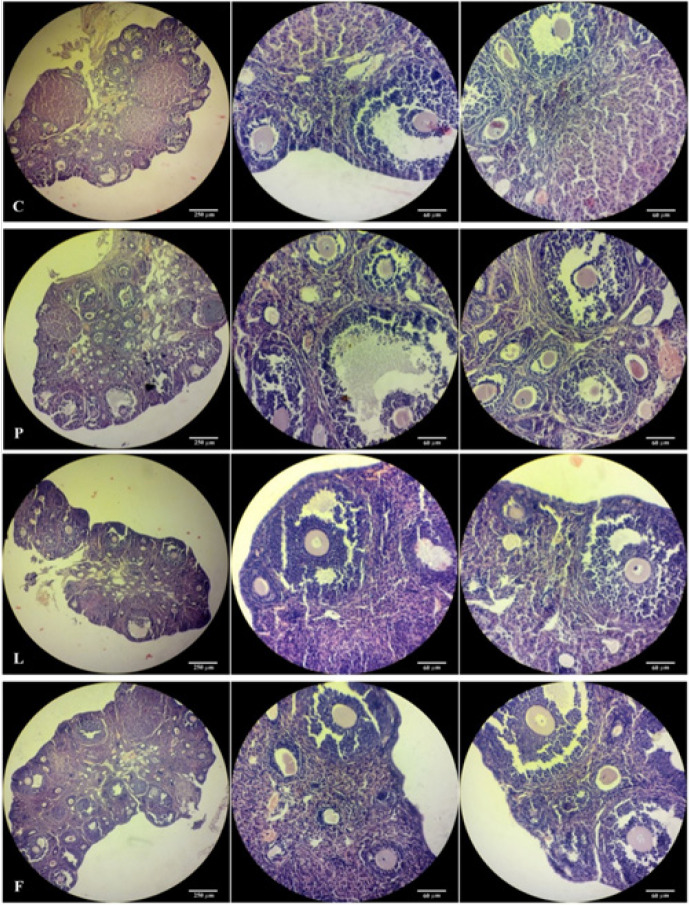
Photomicrographs of the ovarian sections of control (C), PCOS (P), Lupeol (L), and Flutamide (F) treated mice

The size of ovaries in the P group was higher than that of the C group, which was accompanied by a decrease in the number of corpora lutea. Also, an increase of the atretic follicles–having alterations in the shape and size of oocytes, pyknotic nuclei, detached zona pellucida, and formation of precocious antrum along with fragmentation of granulosa cells– is especially evident in secondary and tertiary follicles at higher magnification. Improvement in the histopathologic status of the ovaries in the L and F groups is associated with a decrease in the number of damaged and atretic follicles and an increase in the number of corpora lutea (Figure 3).

The total count of follicles as a quantitative measure of the histological status (Table 1) showed a significant decrease in the number of intact primary, secondary, tertiary, and graafian follicles and by contrast a significant increase in the number of atretic and morphologically injured primary, secondary and tertiary follicles in the P group compared to the control group. Also, the number of corpora lutea in the P group was significantly (*P*<0.05) reduced compared to the C group. In groups L and F, compared to group P, follicles were histologically improved and the number of healthy follicles remarkably increased at all stages of development. Moreover, the rate of follicular atresia in the L and F groups decreased. There was an insignificant increase in the number of corpora lutea in the L group, compared to the P group.

Comparing the L and F groups, the number of primary follicles in group L was significantly higher than in the F group, and the number of healthy follicles in the F group was significantly higher than in the L group. The number of follicles in other growth and developmental stages as well as the number of corpora lutea between the two groups did not show any significant difference (*P*>0.05).

***Lupeol and flutamide reduced the PCOS-induced fibrosis in the Ovary***

**Figure 4:**
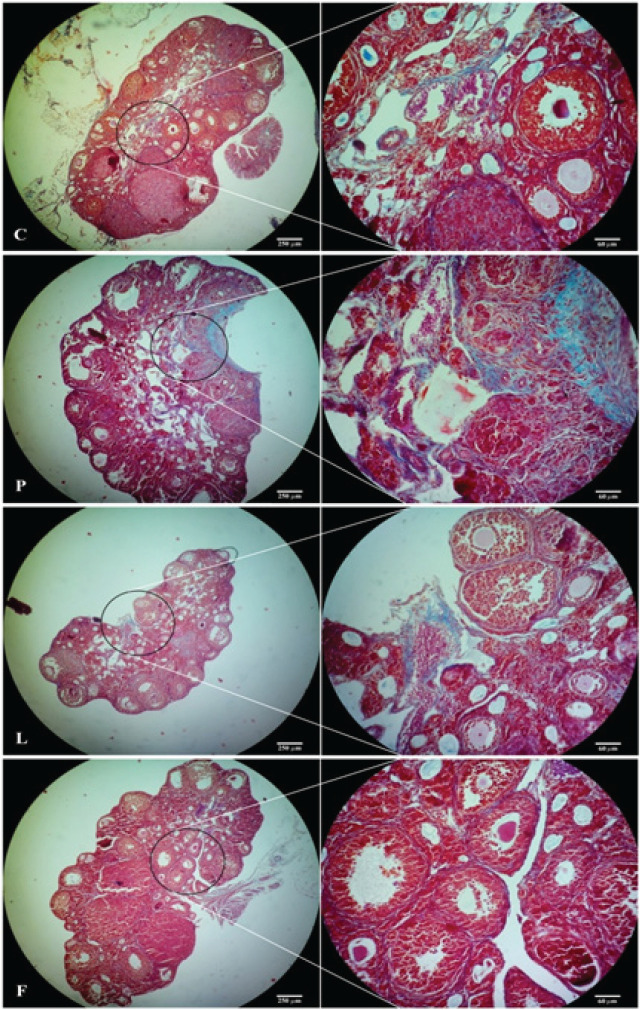
Photomicrograph of the ovarian sections of control (C), PCOS (P), Lupeol (L), and Flutamide (F) treated mice

Photomicrographs of the ovarian sections stained with the Mallory-AZAN method are shown in Figure 4. In the control group, thick fibers of collagen were observed in the medullary stroma and around the blood vessels, and delicate fibers of collagen in the cortex and between the follicles, as well as theca cells layer and around the corpora lutea. In the P group, an increase in the thickness and dispersion of collagen fibers, especially in the cortical stroma and around the follicles was evident as an increased scatter of blue color related to collagen fibers. In the L and F groups, the intensity of the blue color and thickness of collagen fibers were remarkably decreased compared to the P group.

***Quantitative evaluation of fibrosis in ovarian sections by analyzing images***

The amount of collagen fibers in the photomicrographs of the ovarian sections, quantified through image analysis, using Matlab software (V. 8.1.0) are shown as a percentage of the blue pixels to the entire image pixels (Table 2). The results showed that the percentage of tissue collagen in group P was significantly (*P*<0.05) higher than that of group C and was significantly decreased in the L and F groups compared to the P group (*P*<0.05). There was no significant (*P*<0.05) difference between L and F groups. 

***Oxidative stress assessment***

*Total anti-oxidant capacity (TAC) of the ovarian tissue and blood serum*

**Figure 5.**
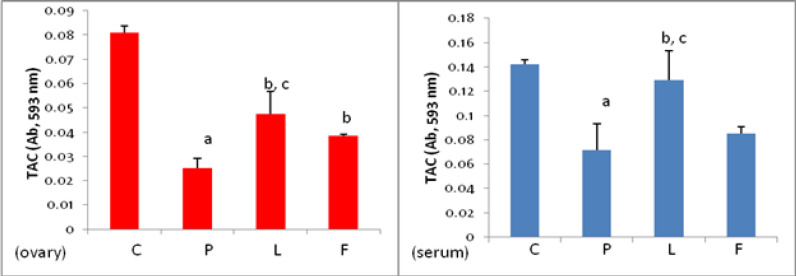
Total anti-oxidant capacity of ovarian tissue and serum samples in control (C), PCOS (P), Lupeol (L) and Flutamide (F)-received mice

As shown in Figure 5, the total anti-oxidant capacity of ovarian tissue and blood serum in group P significantly decreased compared to the control group (C). Treatment with lupeol resulted in a significant increase in this parameter in ovarian and serum samples of group L compared to group P. Treatment with flutamide resulted in an increase in TAC in group F compared to group P, which was statistically significant in ovarian tissue but not in the serum sample. Comparison of TAC changes between L and F groups shows that the effect of lupeol on TAC increase in ovarian tissue and serum is significantly greater than that of flutamide (*P*<0.05).

*Lupeol and flutamide lowered the lipid peroxidation in ovarian tissue and serum*

**Figure 6:**
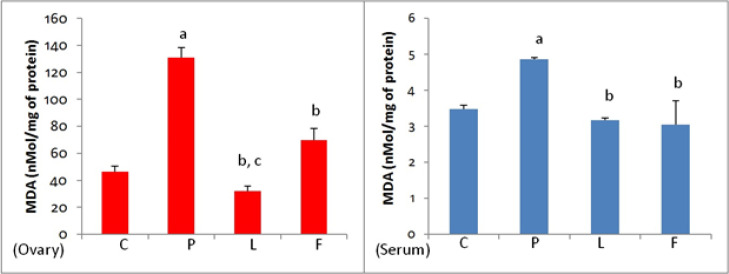
**. **Effect of Lupeol and Flutamide on MDA concentration in ovarian tissue and serum of the control (C), PCOS (P), Lupeol (L) and flutamide (F)-treated mice

The amount of MDA in ovarian tissue and serum samples of group P was significantly (*P*<0.05) higher than that of group C, while treatment with lupeol and flutamide reduced the PCOS-induced lipid peroxidation rate significantly (*P*<0.05) both in ovarian tissue and serum. Comparison between the L and F groups showed that MDA concentration in the ovarian tissue of group L was significantly lower than group F (*P*<0.05), but there was no significant difference in serum levels between these two groups (Figure 6).

*Lupeol reduced the PCOS-elevated protein carbonylation in the ovary and serum*

**Figure 7:**
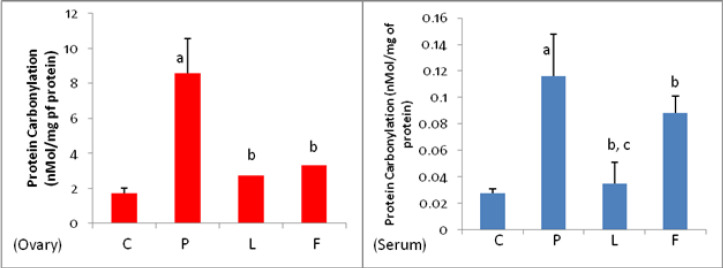
Effect of Lupeol and Flutamide on protein carbonylation in ovarian tissue and serumof the control (C), PCOS (P), Lupeol (L) and flutamide (F) -received mice

As presented in Figure 7, the protein oxidation assessment revealed that the experimentally-induced PCOS resulted in significant protein carbonylation both in the ovary and serum. Lupeol treatment reduced significantly the protein oxidation in ovarian tissue and blood serum of animals in group L. Flutamide was also able to reduce the concentration ​​of this parameter in group F. Comparison of protein carbonylation rate between groups L and F showed that there was no significant difference between L and F in ovarian tissue, however in serum, the level of carbonylated protein in group L was significantly lower than in group F (*P*<0.05).

***Lupeol enhanced the PCOS-reduced fertility rate in the in vitro fertilization (IVF) assessment***

**Figure 8:**
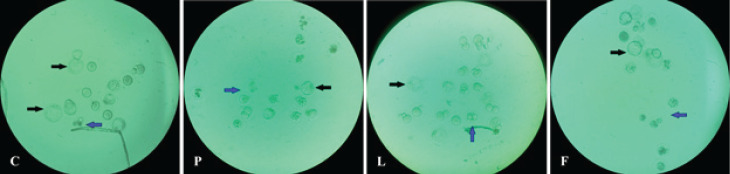
Photomicrographs of *in vitro* fertilized embryos of mice in groups C, P, L and F after 5 days incubation

The results of *in vitro* fertilization and embryonic growth in all four groups are shown in Figure 8 and quantified data are presented in Table 3. The total number of oocytes per mouse and the relative number of top-quality oocytes in group P showed a significant decrease compared to the control group. The fertilization rate in group P was 83.33%, which is significantly (*P*<0.05) lower than that of group C (95.65%). The fertilization rate in group L was 94.66%, which was significantly increased in comparison with group P. In group F, however, an 85.9% fertilization rate is indicating no significant difference from group P. Comparison between the two groups of L and F showed that the fertilization rate in group L was significantly higher than that in group F (*P*<0.05). 

The percentage of two-cell embryos as a marker for initiation of cleavage showed that in group P (64.0%) it was significantly lower than that of group C (90.91%). In groups L (74.65%) and F (77.61%), the two-cell embryo was significantly higher than in group P (*P*<0.05). Comparison between groups L and F did not show any significant difference between the rates of two-cell embryo development.

The percentage of blastocyst formation in group C was 60.91%, whereas in group P it dropped to 28.0% and it was found to be significantly different from group C. A significant difference was recorded between groups L (39.44%) and F (40.30%) in terms of blastocyst formation compared to group P (*P*<0.05). There was no significant difference between the two groups of L and F in the blastocyst formation rate. 

The mean percentage of arrested embryos in group P was about 72.0%, which was significantly higher compared to group C (39.09%), meanwhile in groups L (60.56%) and F (59.77%), this parameter was significantly decreased (*P*<0.05) and there was no significant difference between the two groups of L and F.

White arrow: nucleated epithelial cell, black arrow: anucleated cornified epithelial cell, white arrow head: polymorphonuclear leukocytes.

a: significant difference (*P*<0.05) compared to group C, b: significant difference (*P*<0.05) compared to group P, c: significant difference (*P*<0.05) between L and F groups.

Increasing the ovarian size, decreasing the number of corpora lutea, and increasing the atretic follicles with the formation of premature cavities and granulosa cell fragmentation are evident in group P

(H & E staining, X100, and X 400 enlargements)

**Table 1:** **. **The Ovarian follicle count at different developmental stages in: Control (C), PCOS (P), Lupeol (L) and Flutamide (F) recieved mice

	Follicle								corpus luteum	cystic follicle
	primary		secondary		tertiary		Graafian			
	healthy	atretic	healthy	atretic	healthy	atretic	healthy	atretic		
C	163.7±11.7	7/0±2.3	53.6±13.5	95.0±10.4	18.3±4.2	14.7±2.8	1.7±0.5	4.5±1.0	9.3±1.5	0
P	143.0±6.1^a^	33.5±12.7^a^	37.8±6.2^a^	164.0±15.4^a^	11.2±3.1^a^	49.6±4.2^a^	1.1±0.6^a^	7.7±1.7^a^	3.0±1.0^a^	0
L	153.3±5.9^b^	17.7±4.6^b,c^	48.5±3.5^b^	138.2±10.2^b^	16.7±2.0^b^	21.3±3.5^b^	2.2±0.5^b^	4.7±1.3^b^	4.3±1.5^b^	0
F	161.3±1.7^b^	11.6±2.8^b,c^	58.7±4.2^b^	122.0±23.6^b^	20.0±4.8^b^	18.3±4.2^b^	2.3±0.6^b^	4.5±0.8^b^	4.7±0.6^b^	0

a: significant difference (*P*<0.05) compared to group C, b: significant difference (*P*<0.05) compared to group P, c: significant difference (*P*<0.05) between L and F groups

**Table 2:** **. **Percentage of ovarian collagen according to quantitative image analysis by Matlab (V. 8.1.0) in the Control (C), PCOS (P), Lupeol (L) and Flutamide (F)-treated mice

Groups	Percent of tissue collagen (Mean ± SEM)
C	7.54±2.27
P	17.80±2.65^a^
L	9.14±1.76^b^
F	10.56±2.24^b^

a: significant difference (*P*<0.05) compared to group C, b: significant difference (*P*<0.05) compared to group P

Increasing the thickness and dispersion of connective tissue fibers in cortical stroma of the ovary and between the follicles is evident in the group P. (AZAN staining, X100 and X400 magnification).

a: significant difference compared to group C (*P*<0.05), b: significant difference compared to group P (*P*<0.05), c: significant difference (*P*<0.05) between L and F.

a: significant difference compared to group C, b: significant difference compared to P, c: significant difference between L and F (*P*<0.05).

a: significant difference compared to group C, b: significant difference compared to P, c: significant difference between L and F (*P*<0.05).

Black arrow: healthy blastocysts, blue arrow: arrested embryo. (Magnification: X100).

**Table 3:** Quantitative presentation of oocytes number, quality, fertilization and embryo development rates in the control (C), PCOS (P), Lupeol (L) and flutamide (F)-treated mice

Group	Mice (N)	Retrieved oocyte (N)	Top quality oocytes (N)	Low quality oocyte (N)	Fertility (%)	2 Cell Embryo	Blastocysts	Arrested embryo	type I	type II	type III
C	10	115	115 (100%)	0 (0%)	110 (95.65%)	100 (90.91%)	67 (60.91%)	43 (39.09%)	0 (0%)	3 (2.73%)	40 (36.36%)
P	10	72^ a^	60^ a^ (83.33%)	12^ a^ (16.67%)	50^ a^ (83.33%)	32^ a^ (64%)	14^ a^ (28.0%)	36^ a^ (72.0%)	7 (14.0%)	6 (12.0%)	23 (46.0%)
L	10	83^ b^	75^ b^ (90.36%)	8^ b^ (9.64%)	71^ b,c^ (94.67%)	53^ b^ (74.65%)	28^ b^ (39.44%)	43^ b^ (60.56%)	3 (4.23%)	3 (4.23%)	37 (52.11%)
F	10	85^ b^	78^ b^ (91.76%)	7^ b^ (8.24%)	67^ c^ (85.90%)	52^ b^ (77.61)	27^ b^ (40.30%)	40^ b^ (59.70%)	3 (4.48%)	5 (7.46%)	32 (47.76%)

a: significant difference compared to group C, b: significant difference compared to P, c: significant difference between L and F (*P*<0.05)

## Discussion

PCOS is a multi-figural syndrome with abnormalities in the function (anovulation or oligovulation, hyperandrogenism) and structure (decreased follicular development, increased ovarian volume) of the ovary. Several mechanisms including elevation of inflammatory biomarkers, hormonal disturbances, and oxidative stress are among the well-established molecular mechanisms of PCOS. In the current study, DHEA-induced PCOS in mice met the above-mentioned criteria, and experimentally-induced PCOS was characterized by increased atretic and fibrotic follicles, elevated concentration of testosterone, and remarkable enhancement of oxidative stress. Additionally, the outcome of IVF using the oocytes from PCOS-positive mice resulted in a significantly declined number of blastocysts and two-cell embryos. Both tested compounds of lupeol and flutamide were able to reduce the DHEA-induced PCOS-related disorders.

The primary point in this study should be confirming the used methodology for PCOS induction, and indeed our results from a morphological point of view to functional tests such as hormonal disturbances and induced oxidative stress confirmed the DHEA-induced PCOS in mice. 17-ketoestroids are the main precursors of all potent sex steroids in the gonads and adrenal glands. Injected DHEA mimics the condition that happens due to increases in androgen production by theca cells as a consequence of increased LH pulse frequency. At the same time, the lower FSH levels result in abnormal follicle maturation and consequently anovulation (47). Another fact in the pathophysiology of PCOS due to hyperandrogenemia is that increased androgens (DHEA) cause desensitization of the hypothalamus to progesterone/estrogen negative feedback that in turn elevates both ovarian androgen production and gonadotropin secretion, too (48). Based on consensually accepted diagnostic criteria, clinical and/or biochemical signs of hyperandrogenism have also been counted as some of the main criteria for PCOS confirmation (49). It has been well documented that androgens via androgen receptors enhance FSH-stimulated follicular differentiation and consequently inhibit follicular maturation, supporting the DHEA-induced follicular atresia in our current study. In an *in vitro* study a strong suppression of androgens on FSH-stimulated granulosa cell proliferation by cell cycle arrest at G2/M phase has been reported (50). One of the histopathological findings of our study is that PCOS-positive mice showed an increased production of collagen fibers indicating a fibrotic state. Although fibrosis may have several etiologies, however in most cases a typical chronic inflammation before fibrosis has been reported. Early studies reported that at the final stage of follicle atresia due to the high concentration of LH, theca cells are replaced/transformed with/to fibroblasts, suggesting that the previously described fibrotic state of DHEA-induced PCOS, likely is due to excessive androgen presence (51). An *in vivo* study on rodent model revealed at least partly that the molecular pathway of inflammatory reactions in the PCOS cases mediated via the JNK signaling pathway and therefore using a JNK inhibitor (SP600125) resulted in a remarkable reduction of inflammatory biomarkers including hyperemia, inflammatory cell infiltration, vascular congestion and consequently reduction of fibrotic states characterized by reduced amount of produced collagen type IV in PCOS positive rats (52). Another human study, which was conducted on 171 PCOS-positive women, revealed that the serum levels of IL-1α and IL-17 in the PCOS were significantly higher and lower than those in control cases, respectively, confirming that PCOS is a low-level chronic inflammatory disorder (53).

In this study, we in addition to structural and functional changes evaluated the oxidative status by measuring the various biomarkers. Our findings indicate a remarkable elevation of oxidative stress in the PCOS-induced animals. Oxidative stress or excessive reactive oxygen species (ROS) play a crucial role in the pathogenesis of PCOS via various mechanisms including activation of protein kinases (such as JNK as mentioned earlier) and in turn participation in the regulation of various genes, increasing the release of Ca^2+^ from the endoplasmic reticulum and consequently contributing in the follicular arrest, induction of insulin resistance, and protein, lipid, and DNA oxidation (54-56). 

 The lower percentage of fertility rate in the PCOS-induced mice was another finding of the current study, confirming the previously described structural malformation, hormonal disorder, and abnormal function of fertility-related organs and in particular the ovaries. 

The second part and of course the novel part of this study was devoted to highlighting the ameliorative effects of lupeol on DHEA-induced PCOS in mice models. Our results showed that almost all the studied factors including structural changes, oxidative disturbances, and low rate of IVF outcome were markedly ameliorated in the lupeol-treated animals. The potential effects of lupeol on PCOS-induced changes were compared with the effects of flutamide as a known anti-androgenic compound. 

The current study indeed showed that lupeol at the tested dose was remarkably able to reduce the PCOS-induced alterations in the structure and more substantially in the function of the ovary. As discussed earlier one of the accepted etiologies in the PCOS induction is hyperandrogenism, hence any compound with anti-androgenic capacity will be worth examining as a potential anti-PCOS agent. During the last decade, increasing evidence demonstrated that lupeol in different *in vivo* and also *in vitro* models showed anti-androgenic activities (34, 57). The exact molecular mechanism of lupeol in PCOS cases is not fully understood yet, however, it seems lupeol as a known inhibitor of androgen receptors (AR) more likely competes with DHEA to bind to AR and consequently reduce the PCOS-related structural (atretic follicles and fibrosis) and also functional alterations (alterations in hormonal situation and low outcome of IVF). Previous studies highlighted a few mechanisms for the anti-androgenic activity of lupeol in human androgen receptor-positive LNcap cells as inhibition of activity of transcription factor AR, ducking with AR at ligand-binding site, decreasing the mRNA and protein expression of AR target gene, binding with AR more efficiently, and inhibition of AR signaling (35). 

Another finding of this study is the antifibrotic effect of lupeol in PCOS-positive mice. Although the etiology of fibrosis could vary between different organs, an unequivocal role of the chronic inflammation prior to fibrosis has been consensually accepted. In chronic inflammation in addition to immune cells, the injured cells also contribute to releasing a wide range of inflammatory cytokines including transforming growth factor-β (TGF-β), which provokes inflammation and enhances excessive extracellular matrix production (58). Therefore, to reduce fibrosis in advance, at least partly if not all, one of the key ways could be attenuation of chronic inflammatory reactions. In this regard, one of the most studied properties of lupeol is its anti-inflammatory capacity. The anti-inflammatory effects of lupeol have been reported in mice models via reduction of iNOS-presenting cells and also through the inhibition of myeloperoxidase release from human neutrophils (59).

Among others, excessive oxidant generation and obesity condition are the other two risk factors in the incidence of PCOS. Our results in this study showed that lupeol can reduce the oxidative stress biomarkers. This finding is supported by previous studies as the anti-oxidant and anti-hypercholesterolemia effects of lupeol frequently have been documented (32, 60). 

## Conclusion

Taken together, our study showed that the experimentally-induced PCOS in rodent model could be a valuable laboratory model for mimicking at least partly the real PCOS in human beings and allows studying the anti-PCOS effects of potential agents. At the same time, our results also highlighted that lupeol as a dietary triterpenoid showed ameliorative effects on PCOS, which are characterized by improvement in the PCOS-induced structural and functional factors and remarkable anti-oxidant capacity. 

## Authors’ contributions

A RG performed experiments, analyzed the data, and drafted the manuscript; A A helped perform experiments and data analyses; R S reviewed the manuscript; H M designed and performed the experiments, drafted, revised, and discussed the manuscript. The finalized manuscript was approved by all authors.

## Ethical Approval

This study was approved by the Urmia University Ethical Committee (IR-UU-AEC/1395/89/2d).

## Conflicts of Interest

The authors declare no conflicts of interest.
